# Differential gene expression in *Drosophilamelanogaster* and *D.nigrosparsa* infected with the same *Wolbachia* strain

**DOI:** 10.1038/s41598-021-90857-5

**Published:** 2021-05-31

**Authors:** Matsapume Detcharoen, Martin P. Schilling, Wolfgang Arthofer, Birgit C. Schlick-Steiner, Florian M. Steiner

**Affiliations:** Molecular Ecology Group, Department of Ecology, University of Innsbruck, Technikerstr. 25, 6020 Innsbruck, Austria

**Keywords:** Evolutionary ecology, Molecular ecology, Pathogens, Symbiosis

## Abstract

*Wolbachia* are maternally inherited endosymbionts that infect nearly half of all arthropod species. *Wolbachia* manipulate their hosts to maximize their transmission, but they can also provide benefits such as nutrients and resistance against viruses to their hosts. The *Wolbachia* strain *w*Mel was recently found to increase locomotor activities and possibly trigger cytoplasmic incompatibility in the transinfected fly *Drosophila* *nigrosparsa*. Here, we investigated, in females of both *D.* *melanogaster* and *D.* *nigrosparsa*, the gene expression between animals uninfected and infected with *w*Mel, using RNA sequencing to see if the two *Drosophila* species respond to the infection in the same or different ways. A total of 2164 orthologous genes were used. The two fly species responded to the infection in different ways. Significant changes shared by the fly species belong to the expression of genes involved in processes such as oxidation–reduction process, iron-ion binding, and voltage-gated potassium-channel activity. We discuss our findings also in the light of how *Wolbachia* survive within both the native and the novel host.

## Introduction

Different gene expression patterns, shown by tools such as quantitative PCR, microarrays, and high-throughput RNA sequencing, reveal how organisms respond to different environments. RNA sequencing is a powerful method to study differential gene expression because it can detect a whole gene expression across particular tissues^1^. Recently, differential gene expression analysis using RNA sequencing has been widely used to study relationships between hosts and their endosymbionts; examples include dynamics of gene expression across host life cycles^2,3^ and endosymbiont influence on host speciation^4^.

*Wolbachia* (Alphaproteobacteria) are a group of bacterial endosymbionts found in arthropods and nematodes^5^. It is estimated that around half of all arthropod species are infected with *Wolbachia*^6,7^, with their diversity estimated at around 100,000 strains^8^. Maternally transmitted, these endosymbionts manipulate their host's reproduction for their benefits in various ways, such as feminization, cytoplasmic incompatibility, male-killing, and parthenogenesis^5^. In some cases, *Wolbachia* also provide benefits to their hosts, such as by supplying vitamins to *Cimex* *lectularius* bedbugs^9^ and providing virus protection in *Drosophila* species^10–13^.

The same *Wolbachia* strain can have different effects in different genetic backgrounds within and across host species^14,15^. A recent study in three black fly species in the genus *Simulium* found differential *Wolbachia* prevalence among species, suggesting host-specific interactions^16^. Additionally, failure to transinfect *Wolbachia* strains from their native hosts to other species has been shown in many species, for example, between related species of parasitic wasps in the genus *Trichogramma*^17^ and *w*MelCS and *w*MelPop from *D.* *melanogaster* to *D.* *nigrosparsa*^18^. However, the mechanisms behind these interactions are not clear.

Microinjection has been used to facilitate transinfection of endosymbionts from one to another host species which facilitates studying effects of the endosymbionts on new hosts. In *Drosophila*, microinjection has been used in several host-endosymbiont studies; for example, to transfer *Wolbachia* *w*MelPop from *D.* *melanogaster* to *Aedes* *aegypti*^19^ and *Aedes* *albopictus*^20^, *w*MelPop from *D.* *melanogaster* to *Drosophila* *simulans*^21^, and *Spiroplasma* between *Drosophila* *hydii*^22^ and *D.* *melanogaster*^23^.

There are around 2000 *Drosophila* species^24^ ranging from habitat generalists to habitat specialists^25^. The model organism *Drosophila* (*Sophophora*) *melanogaster* is a generalist with a cosmopolitan distribution^26^. *Drosophila* (*Drosophila*) *nigrosparsa* is an alpine species found at around 2000 m above sea level in European mountain systems^26^. Due to the habitat specificity of *D.* *nigrosparsa*, molecular and physiological traits and potential effects of warming temperatures on this species have been studied^27–33^. Wild populations of *D.* *melanogaster* are commonly infected with *Wolbachia*^34^, while no wild population of *D.* *nigrosparsa* infected with *Wolbachia* has been found to date (M. Detcharoen, unpubl.). We recently transinfected the *Wolbachia* strain *w*Mel from *D.* *melanogaster* into *D.* *nigrosparsa* and studied several traits including *Wolbachia* density as well as host temperature tolerance, larval and adult locomotion, and cytoplasmic incompatibility^18^. Our analysis of *D.* *nigrosparsa* infected with *Wolbachia* *w*Mel revealed increased locomotion compared with flies cured from their infection as well as hints of weak cytoplasmic incompatibility^18^.

In searching for molecular mechanisms behind the increased locomotion and cytoplasmic incompatibility in *D.* *nigrosparsa*, we here used differential gene expression analysis by RNA sequencing. In a comparative analysis, we aimed to investigate the effects of *Wolbachia* *w*Mel on gene expression in *D.* *melanogaster*, the native host, and *D.* *nigrosparsa*, the novel host, to find out whether these two species respond to the infection in the same or in different ways.

## Experimental procedures

### Fly culture

*Drosophila* *melanogaster* isofemale line w1118 (provided by Luis Teixeira) naturally infected with *Wolbachia* strain *w*Mel was divided into three infected lines, that is, mi_1, mi_2, and mi_3, upon arrival at the laboratory of the Molecular Ecology Group at the University of Innsbruck (Austria). The uninfected *D.* *melanogaster* line mu_0 (likewise provided by Luis Teixeira) had been created by treating the infected isofemale w1118 line with tetracycline hydrochloride to remove *Wolbachia* more than ten years before the start of this study^10^. The infected lines and the uninfected line had been maintained at 19 °C in the laboratory in Innsbruck for 30 and six generations prior to the experiment, respectively.

The uninfected *D.* *nigrosparsa* (nu_0) used in this study originated from the isofemale line iso12. This line was established using a population from Kaserstattalm, Tyrol, Austria (47.13° N, 11.30° E) in 2010^30,32^ and had been maintained in the laboratory for about 60 generations before the founding of line nu_0. *w*Mel infection of *D.* *nigrosparsa* was achieved as described in Detcharoen et al.^18^. Briefly, cytoplasm of *D.* *melanogaster* containing *Wolbachia* *w*Mel was transinfected into embryos of *D.* *nigrosparsa* line nu_0, and three infected lines, ni_3, ni_6, and ni_8, were generated.

Both fly species were cultured as described in Kinzner et al.^27^. Briefly, *D.* *melanogaster* was cultured using corn food at a density of 80 embryos per vial with 8 ml food. For *D.* *nigrosparsa*, approximately 50 males and 50 females were put in a mating cage supplied with grape-juice agar, malt food, and live yeast. Embryos and larvae were collected and put in glass vials with 8 ml malt food at a density of 80 embryos per vial. All fly stocks were maintained at 19 °C, 16 h : 8 h light : dark cycle and 70% relative humidity in an incubator (MLR-352H-PE, Panasonic, Japan).

The two *Drosophila* species used have different development times, that is, around 20 days for *D*. *melanogaster* and 60 days for *D.* *nigrosparsa* from eggs to adults at 18–19 °C^27,35^. Five-day old *D.* *melanogaster* were considered mature and have been used as such in a wide range of studies, including transcriptomic studies^36–38^. For *D.* *nigrosparsa*, oviposition activity was found to start at an age of seven days, and the egg-laying activity to increase during the second week after eclosion^18,27^; thus, 14-day old *D.* *nigrosparsa* were used here. In *D.* *melanogaster* and *D.* *nigrosparsa*, *Wolbachia* density starts to peak at ages of around five and 14 days, respectively^18,39^. In addition, it was previously found that variation in *Wolbachia* density in the *D.* *nigrosparsa* older than 14 days was much higher than in flies at the age of 14 days^18^. Thus, gene expression of the two fly species was compared at the same physiological stage regardless of age because of the different development times. This approach has been used in comparative transcriptomics in multi-species studies of various insect orders^40,41^.

Five females per line were randomly collected before the experiment to check for *Wolbachia* infection. DNA was extracted, and the infection of each line in the generation used for RNA analyses was confirmed using PCR with the primers wsp81F and wsp691R^42^. Besides the infection of lines in both species with *Wolbachia*, all fly lines used in this study were found not to be infected by *Cardinium*, *Spiroplasma*, and *Rickettsia* (data not shown).

### RNA extraction and sequencing

Fourteen-day old females of *D.* *nigrosparsa* and 5-day old females of *D.* *melanogaster* of both uninfected and infected lines (totaling eight lines) were randomly collected using short carbon-dioxide anesthesia. Only female flies were used because *Wolbachia* are maternally transmitted and because changes in locomotion and cytoplasmic incompatibility due to *Wolbachia* were found in *D.* *nigrosparsa* females^18^. All fly lines were killed by snap freezing in liquid nitrogen after taking them from their regular regimes as described in the previous section and at the same time of the day (at 11 a.m.) to control for circadian-rhythm based variation in gene expression. RNA from individual flies was extracted using RNeasy Micro Kit (Qiagen, Hilden, Germany) following the manufacturer’s protocol, including removing of DNA step using DNase I. Quantity of RNA was measured using Quant-iT RiboGreen RNA Assay Kit (Thermo Fisher Scientific, USA). RNA extracts of five individuals belonging to the same line were pooled to have a minimum RNA content of 2.5 µg per replicate; five replicates per fly line were established totaling 40 replicates. RNA library preparations, including removing of ribosomal RNA and 75-bp single-end sequencing using Illumina NextSeq 500, were done at IGA Genomics (Udine, Italy). Briefly, ribosomal RNA was removed, adaptors were ligated to RNA fragments, and libraries were prepared using TruSeq Stranded Total RNA (Illumina, USA).

### Sequence alignment and differential expression analyses

Single-end raw reads (SRA database, BioProject PRJNA602188, BioSample SAMN13885146-SAMN13885185) were subjected to quality-check with FastQC version 0.11.8 (http://www.bioinformatics.babraham.ac.uk/projects/fastqc). Trimmomatic version 0.38^43^ was used to remove adapters including the first and the last three nucleotides, to cut reads when the average Phred score dropped below 20 in a 4-bases sliding window, and to drop reads shorter than 40 bases. Reads were quantified relative to their reference transcriptomes using Salmon version 0.12.0^44^, that is, reads belonging to *D.* *melanogaster* were mapped to the reference transcriptome of *D.* *melanogaster* build BDGP6, and reads belonging to *D.* *nigrosparsa* were mapped to its previously published transcriptome^32^. This reference transcriptome of *D.* *nigrosparsa* was filtered and annotated against the transcriptomes of twelve *Drosophila* species. The *D.* *nigrosparsa* reference transcriptome is based on RNA from males and females of all life stages, has 18,016 transcripts that belong to 7197 loci, and a size of 53 Mb. The numbers of quantified reads were imported to R version 4.00^45^ using the package tximport version 1.14.0^46^.

The genes of *D.* *nigrosparsa* with at least one read mapped to them were translated to protein sequences and blasted to *D.* *melanogaster* using tblastn function implemented in Flybase^47^ to search for orthologous genes. Orthologous genes found were rechecked against the published *D.* *nigrosparsa* data^32^. Each orthologous gene of both species was checked again for expression profile on Flybase’s RNA-Seq Expression Profile. In this step, samples with many genes found to be only expressed in males were suspected to be male-contaminated and thus were removed from all further analyses. Exclusively orthologous genes found in both *D.* *melanogaster* and *D.* *nigrosparsa* were used as they allow a direct comparison between infected and uninfected individuals of each species. Genes with less than 50 counts across all samples were removed. BaySeq version 2.16.0^48^ was used to analyze differential expression between uninfected and infected flies of each species. Priors were estimated using a negative binomial distribution with quasi-maximum likelihood, and posterior likelihoods for each orthologous gene were calculated. Genes were sorted by their posterior likelihood, and those in the upper quartile were selected. A list of the differentially expressed genes was used as input and the list of the default *D.* *melanogaster* genes as background for Gene Ontology (GO) analyses using DAVID version 6.8^49^, and the Benjamini–Hochberg procedure was used to control for false-discovery rate using an alpha value of 0.05. Normalized read counts of genes with posterior probabilities greater than 0.5 were grouped based on Pearson correlation and visualized with heatmaps generated using the R package NMF^50^. The R package vegan version 2.5-6^51^ was used to calculate analysis of similarities (ANOSIM) among samples regarding infection status, lines, and species and non-metric multidimensional scaling (NMDS) with Bray–Curtis dissimilarities to visualize the similarities based on read counts of both species. We additionally used DESeq2 version 1.30.1^52^ to compare the results analyzed with BaySeq. All analyses were done in R version 4.0.0^45^, and visualizations were created using the package ggplot2^53^.

## Results

After quality control, averages of (mean ± standard deviation) 24.8 ± 7.1 and 23.4 ± 4.9 million high-quality reads were found per replicate (each representing five individuals pooled before RNA-seq) of *D.* *melanogaster* and *D.* *nigrosparsa*, respectively. About 85% of the *D.* *melanogaster* reads and 80% of the *D.* *nigrosparsa* reads were mapped to their reference transcriptomes. We removed the uninfected *D.* *melanogaster* replicate sample mu_0.1 from the analyses because of a contamination with male flies. A total of 2164 genes in *D.* *nigrosparsa* were found to be orthologous with genes in *D.* *melanogaster*. After removing genes with low expression across samples, 2084 genes remained in the dataset. We did not identify *Wolbachia* gene expression in this study because we did not get enough *Wolbachia* sequence in the *D.* *nigrosparsa* samples, and thus exclusively host gene expression was considered.

### Variation among pools of individuals within replicate lines and among replicate lines within species

**Figure 1:**
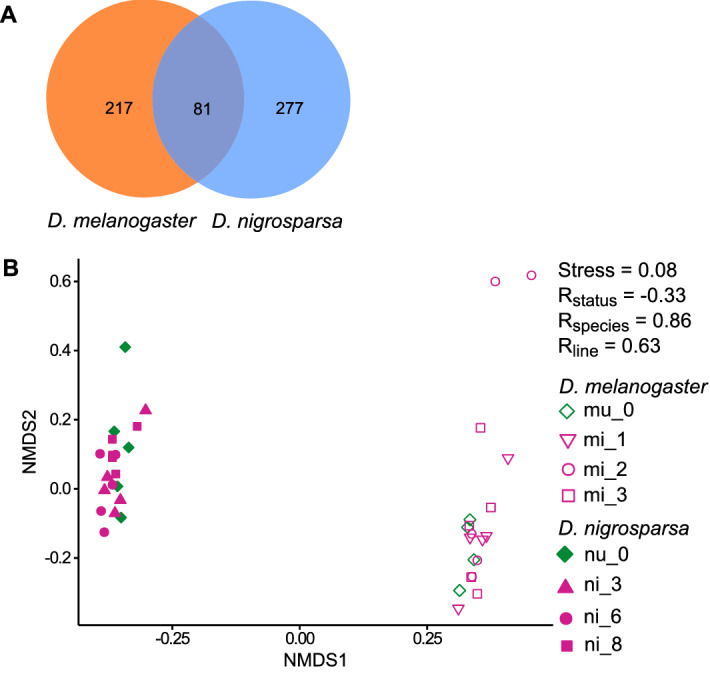
(**A**) Venn diagram of orthologous genes with posterior likelihoods in the fourth quartile of *Drosophila* *melanogaster* and *Drosophila* *nigrosparsa*. Unique genes and genes shared between species are shown. (**B**) Non-metric multidimensional scaling (NMDS) plot using Bray–Curtis dissimilarities with square root transformation of the uninfected and infected samples of both species. Stress (standardized residual sum of squares) values of NMDS and R-values of each group comparison calculated from ANOSIM (Rstatus is between uninfected and infected lines, Rline is among lines, and Rspecies is between species) are shown. For each sample, n = 5, except for mu_1 (n = 4).

Following differential expression analysis, differentially expressed genes between uninfected and infected individuals within species were ordered according to their posterior probabilities (Supplementary Table S1). Most orthologous genes of both fly species had low posterior probabilities. There were 101 genes with posterior probability > 0.5, and there were 298 *D.* *melanogaster* genes in the fourth quartile. Eighty-five genes had posterior probability > 0.5 in *D.* *nigrosparsa*, and 358 genes were in the fourth quartile of *D.* *nigrosparsa* (Supplementary Tables S1 and S2). Among these, 81 genes were found in the two species (Fig. 1A, Supplementary Table S3). Many genes that are in the fourth quartile help in binding and catalytic activities. In addition, there were genes in the fourth quartile involved in the Toll pathway, part of *Drosophila* immune systems, namely Spn47C in *D.* *melanogaster* and Spn47C and Myd88 in *D.* *nigrosparsa* (Supplementary Tables S4 and S5).

**Figure 2:**
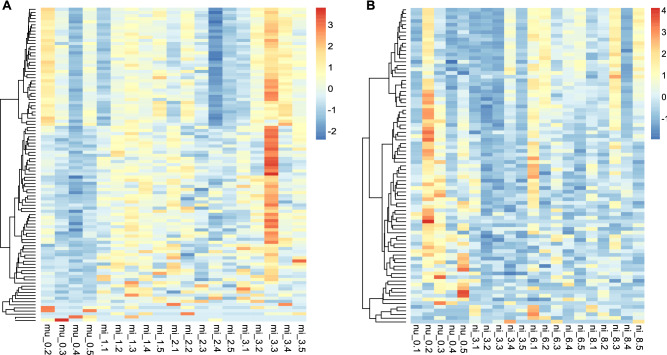
(**A**) Gene expressions of *Drosophila* *melanogaster* uninfected (mu_0) and infected (mi_1, mi_2, and mi_3) and *Drosophila* *nigrosparsa* (**B**) uninfected (nu_0) and infected (ni_3, ni_6, and ni_8). Genes were clustered using Pearson correlation. Only orthologous genes with posterior likelihoods greater than 0.5 were selected (101 and 85 genes of *D.* *melanogaster* and *D.* *nigrosparsa*, respectively). The heatmaps were generated using the R package NMF^50^.

There was strong variation in overall expression pattern among pools of individuals but not between infection status within species. For example, the expression of the infected *D.* *melanogaster* sample mi_3.3 was different from the rest of *D.* *melanogaster* samples. These samples appeared to have more highly expressed genes than the remaining samples (Fig. 2). Some of the differentially expressed genes in both species were expressed more in the infected than in the uninfected flies, while some of them were expressed more in the uninfected flies. Moreover, we observed more variation among lines (ANOSIM, *D.* *melanogaster* R = 0.14, *D.* *nigrosparsa* R = 0.33) than between infection status (ANOSIM, *D.* *melanogaster* R < 0.01, *D.* *nigrosparsa* R = 0.04) (Supplementary Fig. S2).

### Strong difference between species

Gene expression in *D.* *melanogaster* strongly differed from that in *D.* *nigrosparsa*, as calculated with NMDS (Fig. 1B). In the PCA, 80.34% of the variation among orthologous genes in both species were explained by PC1 and the ANOSIM R value of 0.86. In addressing the question about effects of *Wolbachia* on locomotion, we found that BaySeq found several genes that involve in locomotor activities that were more upregulated in infected *D.* *melanogaster*, such as spatzle 5 and beaten path IIb (Supplementary Table S8). In *D.* *nigrosparsa*, we observed a contrasting trend, that is, several genes that involve in locomotor activities, like futsch and tartan, were downregulated in infected compared with uninfected flies.

### Functional analysis of orthologous genes

We used orthologous genes of both species for GO-term analysis. Two GO terms were significantly enriched between infected and uninfected in *D.* *melanogaster* and eight terms in *D.* *nigrosparsa* (Table 1). We found that genes belonging to iron-ion binding (9 of 12 genes, molecular function) and oxidation–reduction process (16 of 20 genes, biological process) were expressed more in the infected than in the uninfected *D.* *melanogaster*. For *D.* *nigrosparsa*, genes of GO terms, such as voltage-gated potassium-channel activity (4 of 4 genes, molecular function), dendrite (5 of 9 genes, cellular component), chitin-based cuticle development (6 of 10 genes, biological process), and integral component of plasma membrane (17 of 28 genes, cellular component) were expressed more strongly in the uninfected than the infected flies, while genes in some GO terms such as olfactory receptor activity and membrane were expressed similarly in infected and uninfected flies (Supplementary Table S6). Two genes were shared between *D.* *melanogaster* and *D.* *nigrosparsa*, belonging to the iron-ion binding, oxidation–reduction process, mitochondrial membrane, and integral component of membrane, namely shd and CG7724. Gene CG7724 was expressed differently in the two species (Supplementary Table S6), in that it was more highly expressed in infected *D.* *melanogaster* than in uninfected lines of the same species, and more in uninfected *D.* *nigrosparsa* than in the infected ones. Only some of the genes in the GO terms in both species had log_2_ fold change bigger than two (Supplementary Table S1).

**Table 1:** Significantly enriched gene ontology terms of *Drosophila* *melanogaster* and *Drosophila* *nigrosparsa*. MF, CC, and BP are molecular function, cellular component, and biological process, respectively.

Species	Category	Term	Name	Genes
*D.* *melanogaster*	MF	GO:0005506	Iron ion binding	Cyp28c1, PH4alphaMP, shd, Cyp28a5, Cyp301a1, Cyp305a1, Cyp318a1, Cyp4g15, Desat2, PH4alphaEFB, Plod, Trh
	BP	GO:0055114	Oxidation–reduction process	CG9503, Cyp28c1, PH4alphaMP, shd, ADPS, CG13078, CG17896, CG6083, CG7724, Cyp28a5, Cyp301a1, Cyp305a1, Cyp318a1, Cyp4g15, Desat2, L2HGDH, Ldh, PH4alphaEFB, Plod, Trh
*D.* *nigrosparsa*	MF	GO:0004984	Olfactory receptor activity	Gr32a, Gr94a, Gr66a, Or92a
		GO:0005249	Voltage-gated potassium channel activity	eag, Elk, Shab, Shal
	BP	GO:0040003	Chitin-based cuticle development	amd, Cpr62Bb, Cpr62Bc, Cpr92F, TwdlV, TwdlY, CG34461, Cht2, Cpr100A, Pcp,
	CC	GO:0030425	Dendrite	Cdk5alpha, futsch, Gr32a, Gr94a, Shal, Cby, Dnai2, Efhc1.1, Gr66a
		GO:0005887	Integral component of plasma membrane	anne, Cad86C, CG11437, CG44098, CG5404, CG8249, DIP-alpha, DIP-delta, Elk, FMRFaR, foi, lbm, Osi2, Osi9, ru, tadr, Tsp42Ek, bdg, CG14691, CG7255, CG7458, CG9706, DIP-beta, mAChR-C, NaPi-III, Osi19, PK1-R, Tre1
		GO:0016020	Membrane	CG10345, CG11437, CG13375, CG2736, CG34056, CG5160, CG8519, CG9444, fz3, Ir93a, Rgk2, CG14767, CG30281, CG3829, CG7255, CLS, DCTN2-p50, Edem1, heix, NaPi-II,I Ras64B, Rep, Rlip, YME1L
		GO:0031966	Mitochondrial membrane	Cyp12b2, dib, shd, Drp1, heix
		GO:0016021	Integral component of membrane	anne, AQP, Ca-alpha1T, Cad86C, CG10073, CG10345, CG13921, CG14234, CG1441, CG14763, CG15385, CG18549, CG2736, CG31637, CG34038, CG4025, CG42566, CG44098, CG5404, CG6707, CG7724, CG8249, CG9231, CG9444, CG9826, Cyp6v1, eag, Efr, FMRFaR, foi, fz3, Gr32a, Gr94a, Hmgcr, inaE, Ir93a, kirre, Krn, lbm, mab-21, mey, MFS9, ND-B16.6, Nep5, Ppcs, ru, Start1, stmASugb, Syx8, tadr, trn, Tsp42Ek, ttv, ZnT77C, bdg, CG10063, CG11007, CG13196, CG13306, CG14232, CG14691, CG14767, CG16953, CG17121, CG2211, CG31229, CG3156, CG34138, CG3829, CG3831, CG6665, CG7255, CG7458, CG7840, CG9536, CG9706, CG9864, CLS, DIP-beta, GC, Gr66a, heix, hrm, loj, mAChR-C, Mco4, Nmda1, Npc1a, Or92a, PK1-R, rtv, sicily, sip3, Syx7, TAF1B, Trc8, Tre1, Ugalt, vir, YME1L

### Results comparisons between different packages

We have compared our results when using BaySeq and when using DESeq2 for two different input datasets, orthologous genes and all the genes of each species. Using orthologous genes, in *D.* *melanogaster*, we found two GO terms with BaySeq but none with DESeq2. In *D.* *nigrosparsa*, we found eight GO terms with BaySeq and nine terms with DESeq2 (Supplementary Table S7). The term membrane is found in both methods. Other terms are related such as plasma membrane (DESeq2) and integral component of plasma membrane (BaySeq).

## Discussion

Our study demonstrated how different *Drosophila* species respond to the same strain of *Wolbachia* by analyzing the expression of orthologous genes shared in *D.* *melanogaster* and *D.* *nigrosparsa*. We found different expression patterns and different genes affected in native host (*D.* *melanogaster*) and novel host (*D.* *nigrosparsa*). We did not check whether there is any change in the *w*Mel genome after transinfection into *D.* *nigrosparsa*. Transinfection may trigger changes in the host genome, but such changes may take place only after a longer time span than used in our study. For instance, changes in the genome of *Wolbachia* were observed 3.5 years after being transinfected into a mosquito cell line but there was no detectable change in the genome once this *Wolbachia* strain had been transinfected into mosquitoes, suggesting adaptation of *Wolbachia* in sensitive environments of cell line^19^. We only used expression data of only orthologous genes found in both *Drosophila* species because we aimed to compare the effect of infection between the two species. It is clear that many genes unique to each species or genes that had not been annotated were not part of the results, and the results might change if all genes were used. We found that gene expression was largely species-specific in *D.* *melanogaster* and *D.* *nigrosparsa*, both when uninfected and infected with the *w*Mel strain of *Wolbachia.* In detail, however, we found a few differences in infection-related gene expression.

In our data, we found variation of gene expression among pools of individuals within replicate lines and among replicate lines within species. With regard to the variation among pools of individuals within replicate lines, gene expression can be influenced by several factors such as the environment and individual variation^54^. For example, around 23 percent of the genes in *D.* *melanogaster* are expressed differently at the individual level, which could be due to individual variation in size or weight^55^. In our study, we cannot evaluate an impact of individual variation as we used a pool of five flies per replicate, but we would perhaps have seen less variation among pools if we had pooled more than five individuals per replicate.

All *D.* *nigrosparsa* used in our study originated from the same isofemale line, which has been maintained in the laboratory since 2010 and in which genetic variation has been greatly reduced. Several studies have used this fly line for genetic and physiological research^18,27,30,32^, with little within-line variation. We pose that the differences between infected and uninfected *D.* *nigrosparsa* we observed are due to effects of *Wolbachia* themselves, not due to the flies’ genetic backgrounds.

We aimed at reducing variation among samples by pooling the flies for sequencing and also normalizing the data for downstream analyses, yet we still observed some variation among the pools and lines. Variation in gene expression among replicate lines might result from several processes such as genetic drift and inbreeding and might be further complicated by selection to the laboratory conditions^56,57^. We also tried to avoid population bottlenecked in our cultures by keeping them at high census sizes, that is, 200 and 100 flies per line of *D.* *melanogaster* and *D.* *nigrosparsa*, respectively. The *D.* *melanogaster* lines used in our study were separated from outbred populations about 2 years before the experiment, whereas each infected *D.* *nigrosparsa* line was derived from a single transinfected female and kept separately for about two years. We suggest that the variation in gene expression among replicate lines may be due to a combination of both drift and inbreeding. In detail, the replicate lines in *D.* *nigrosparsa* were more separated than in *D.* *melanogaster* (Supplementary Fig. S2: R_line_ = 0.33 and 0.14 in values *D.* *nigrosparsa* and *D.* *melanogaster*, respectively), which would be in line with a smaller effective population size due to the stronger bottleneck in *D.* *nigrosparsa* and thus stronger effects of drift.

We found enriched GO terms, iron ion binding and oxidation–reduction process in *D.* *melanogaster*. For the latter GO term, we found that most of the genes were involved in oxidoreductase activities and more strongly expressed in the infected than the uninfected flies. Oxidoreductases produce reactive oxygen species (ROS) as a by-product in the oxidation–reduction process^58^. However, these enzymes can also help in redox homeostasis of the host. *Wolbachia* can help their hosts in the redox homeostasis by producing their own oxidoreductase^59^, which, in turn, reduces the expression of the hosts’ genes.

In *D.* *nigrosparsa*, two GO terms were related to neurons, voltage-gated potassium-channel activity and dendrite. Genes in these two terms were more expressed in the uninfected than the infected flies. We cannot explicitly say that this finding would benefit *D.* *nigrosparsa* because we do not know exactly if genes expressed by *Wolbachia* are more effective than the host’s genes.

Multiple genes belonging to the oxidative phosphorylation process were upregulated in infected *D.* *melanogaster* (Supplementary Table S1). Many genes in these GO terms function as oxidoreductase activators. Oxidoreductases produce reactive oxygen species (ROS) as a by-product in the oxidation–reduction process^58^. However, these enzymes can also help in redox homeostasis of the host. *Wolbachia* can help their hosts in the redox homeostasis by producing their own oxidoreductase^59^, which, in turn, reduces the expression of the hosts’ genes.

The level of ROS has been linked to host immune response to eliminate pathogenic and non-residence bacteria^60,61^*.* In general, *Wolbachia*-infected hosts produce higher ROS level than those cured of *Wolbachia*, for example, in *Drosophila* *simulans* and *Aedes* *aegypti*^62,63^. *Aedes* *albopictus* transinfected with a new *Wolbachia* strain, *w*Mel, expressed more immune genes than when infected with its native strains, *w*AlbA and *w*AlbB, suggesting some co-adaptation between host and endosymbionts^64^. In contrast*,* *Aedes* *polynesiensis* transinfected with *w*AlbB had significant lower ROS than the naturally infected *A.* *polynesiensis* with *w*PolA^65^.

ROS, in addition to being a by-product of molecular respiration, can be produced by heme in its free state^66,67^. Binding and degrading the ROS-induced heme are used in insects to regulate heme homeostasis^67^. Iron is released when the heme is degraded, but an excess of iron is harmful to organisms^68^. *Wolbachia* reduce the iron concentration in host cells by producing proteins that help with iron storage, which as a result changes iron-related gene expression in hosts^69,70^. Here, genes involved in iron-ion binding, which mostly encode proteins that belong to cytochrome P450, were expressed differently in infected and uninfected *D.* *melanogaster* (Supplementary Table S1). Infected *D*. *melanogaster* expressed more of these genes than uninfected ones (Supplementary Table S6).

In *D.* *nigrosparsa*, we found that *Wolbachia* affect genes that belong to olfactory-receptor activity. Half of the genes were expressed more in the infected *D.* *nigrosparsa* flies, and the rest were expressed more in the uninfected flies. Studies found that *w*Ri-infected *D.* *simulans* had better response to food cues, but the response decreased when *D.* *simulans* was transinfected with strains from *D.* *melanogaster*, *w*Mel and *w*MelPop^71,72^. We cannot conclude that infected *D.* *nigrosparsa* have better olfactory activity than the uninfected ones. We cannot conclude that infected *D.* *nigrosparsa* have better olfactory activity than the uninfected ones, but future studies might shed light on that. Our previous results on increased locomotion in infected *D.* *nigrosparsa*^18^ were not supported in the expression pattern of known locomotor genes. While the reasons for this incongruence among studies can be manifold, possible explanations include lack of knowledge of relevant genes in *D.* *nigrosparsa* and temporal lag between gene regulation and effects on the flies and potential circadian rhythms in locomotion patterns.

Several genes of membrane terms were expressed differently between infected and uninfected *D.* *nigrosparsa*. *Wolbachia*, like many endosymbionts, reside within a layer of membrane that is derived from endoplasmic reticulum^73,74^. As *Wolbachia* need to survive within their hosts (e.g., replication and transmission), the ability to make use of host membrane may play a big role in symbiosis with the host^73,75^. In addition, many of these genes also involve in transmembrane transporter activities (e.g., ZnT77C, CG31229, CG9826, and Efr), embryo development (e.g., ru, ttv, CG13196, rtv, mey, and foi), and bitter taste (e.g., Gr32a, Gr94a, and Gr66a). Like in the olfactory terms, we cannot conclude a clear expression pattern of genes in this term.

The threshold we used in our analysis did not result in any significant GO term related to locomotion. However, we found some hints on GO terms that might relate to the locomotion, neuron-related terms, voltage-gated potassium-channel activity and dendrite (Table 1). Two genes within these terms were reported to involve in locomotor activities, namely shal and shab, and low expressions of these genes were reported to cause a locomotion deficit in *D.* *melanogaster*^76–78^. As we found earlier that infected *D.* *nigrosparsa* flies had higher locomotor activities than uninfected ones, we would expect high expression of these genes in the infected flies. However, in this study, these genes were expressed less in the infected than uninfected *D.* *nigrosparsa*. In addition, some other genes involved in locomotion, such as Dh44, ETH, and NPF^79–81^, were found in the fourth quartile of *D.* *nigrosparsa* but not enriched in the GO analysis. Similarly to shal and shab, these genes were expressed more in the uninfected than the infected *D.* *nigrosparsa*.

We believe that the use of a particular software package to analyze RNA-seq data is, at least to some degree, also subjective. Packages differ technically and so do the results they produce, and different authors have different preferences. We did consider multiple software solutions before we did our initial analyses, and in combing through the literature, we found that BaySeq can have higher performance in terms of modeling than other RNA-seq packages^82,83^. Another study^84^ found that BaySeq identified the smallest number of differentially expressed genes compared with other packages and was considered to be the most conservative method. In any case, also those authors found that BaySeq gave the lowest false positive results. In our study, we decided to present the results of the two packages, BaySeq and DESeq2. We found that the results of both packages differ even though not fundamentally. We focus on the results of the BaySeq analyses, but readers interested in the details of the DESeq2 results can find them in Supplementary Table S7. In summary, we found some effects of *Wolbachia* on gene expression to be consistent among lines within species, despite some intraspecific variation. We conclude that the infection by the same *Wolbachia* strain induced a different response in *D.* *melanogaster* and in *D.* *nigrosparsa*. Different gene expression patterns between species can be due to a multitude of factors such as host specificity of the *Wolbachia* strain *w*Mel. A long-term study should be done to observe changes in differential gene expression in *D.* *nigrosparsa*. While we selected physiologically matched stages of female *D.* *melanogaster* and *D.* *nigrosparsa* adult flies, we cannot exclude the possibility that some of the differentially expressed genes could be due to differences in the age of the flies. In addition, our results here were derived from whole-body extraction, but expression in specific tissues such as reproductive tissues should be tested in further studies, for example to observe cytoplasmic incompatibility at the RNA level and to identify how *Wolbachia* reside within these tissues, for a better understanding of *Wolbachia*-induced gene expression.

## Supplementary Information

Supplementary Figures.

Supplementary Tables.

## Data Availability

Reads were deposited at SRA database, BioProject PRJNA602188, BioSample SAMN13885146-SAMN13885185.
