# Supplementary material for: Differential gene expression in Drosophilamelanogaster and D.nigrosparsa infected with the same Wolbachia strain
Source: Sci Rep. 2021 May 31;11:11336. doi: 10.1038/s41598-021-90857-5 (PMC8166886; doi:10.1038/s41598-021-90857-5)
Supplement: Supplementary file 1 — Supplementary Figures. [file 41598_2021_90857_MOESM1_ESM.docx]

**Supplementary figures**

**Differential gene expression in *Drosophila melanogaster* and *D. nigrosparsa* infected with the same *Wolbachia* strain**

Matsapume Detcharoen, Martin P. Schilling, Wolfgang Arthofer, Birgit C. Schlick-Steiner, Florian M. Steiner

Molecular Ecology Group, Department of Ecology, University of Innsbruck


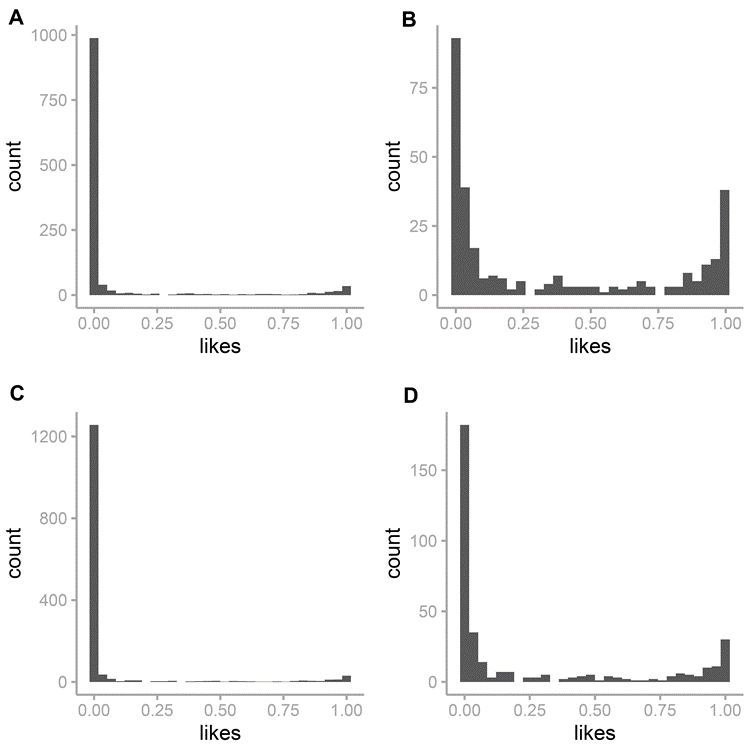


**Figure S1.** Histograms of posterior likelihoods of all orthologous genes and genes in the top quartile in *Drosophila melanogaster* (A and B, respectively) and for *Drosophila nigrosparsa* (C and D, respectively).


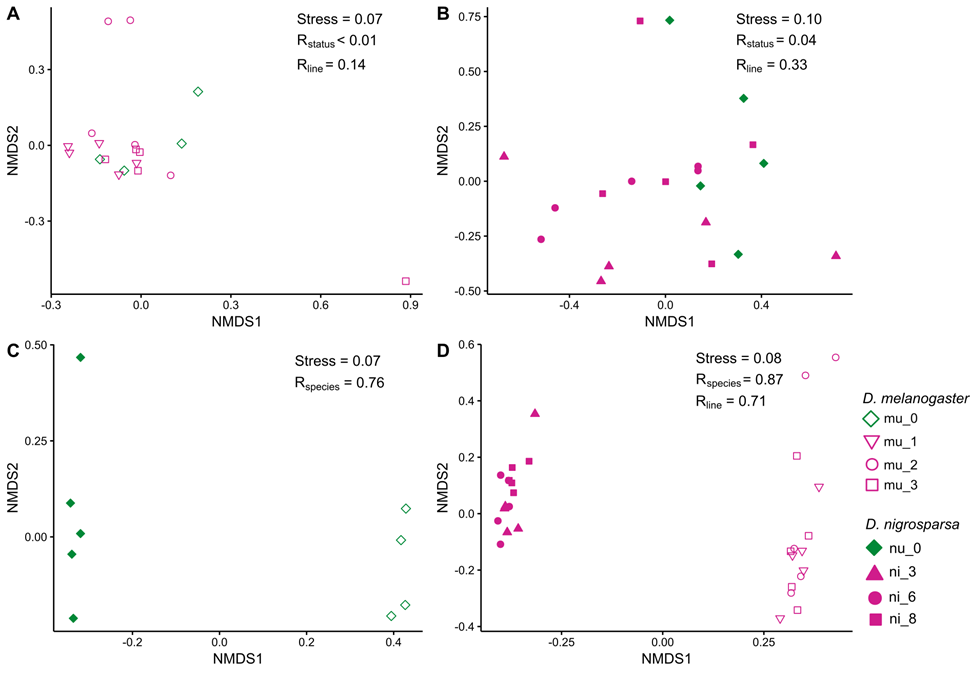


**Figure S2.** Non-metric multidimensional scaling (NMDS) plot using Bray-Curtis dissimilarities with square root transformation used to cluster samples of *Drosophila melanogaster* uninfected (mu_0) and infected (mi_1, mi_2, and mi_3) (A), *Drosophila nigrosparsa* (B) uninfected (nu_0) and infected (ni_3, ni_6, and ni_8), only uninfected samples of both species (C), only infected samples of both species (D). Stress (standardized residual sum of squares) values of NMDS and R-values of each group comparison calculated from ANOSIM (R_status_ is between uninfected and infected lines, R_line_ is among lines, and R_species_ is between species) are shown.
